# Hydroxypropyl-beta and -gamma cyclodextrins rescue cholesterol accumulation in Niemann–Pick C1 mutant cell via lysosome-associated membrane protein 1

**DOI:** 10.1038/s41419-018-1056-1

**Published:** 2018-10-03

**Authors:** Ashutosh Singhal, Lajos Szente, James E. K. Hildreth, Byeongwoon Song

**Affiliations:** 1 Department of Microbiology, Immunology, and Physiology, Meharry Medical College, Nashville, TN 37208 USA; 2 Cyclolab Cyclodextrin Research and Development Laboratory Ltd., H-1097 Budapest, Hungary; 3 Department of Internal Medicine, Meharry Medical College, Nashville, TN 37208 USA

## Abstract

Niemann–Pick type C (NPC) disease is a fatal hereditary neurodegenerative disorder characterized by a massive accumulation of cholesterol in lysosomes and late endosomes due to a defect in intracellular cholesterol trafficking. Dysfunction in intracellular cholesterol trafficking is responsible for about 50 rare inherited lysosomal storage disorders including NPC. The lysosomal proteins NPC1 and NPC2 play a crucial role in trafficking of cholesterol from late endosomes and lysosomes to other cellular compartments. However, the detailed mechanisms of cholesterol trafficking at the late endosomes/lysosomes (LE/LY) are poorly understood. Studies showed that 2-hydroxypropyl-β-cyclodextrin (HPβCD) alleviates the cholesterol accumulation defect in animal model and has been approved for a phase 2b/3 clinical trial for NPC. HPβCD is known to bind cholesterol; however, the mechanisms how HPβCD mediates the exit of cholesterol from the LE/LY compartments are still unknown. Further, another cyclodextrin (CD) derivative, 2-hydroxypropyl-γ-cyclodextrin (HPγCD), was shown to reduce intracellular cholesterol accumulation in NPC patient cells and NPC mice model. Herein, we identified a number of candidate proteins differentially expressed in NPC patient-derived cells compared to cells derived from a healthy donor using a proteomic approach. Interestingly, both HPβCD and HPγCD treatments modulated the expression of most of these NPC-specific proteins. Data showed that treatment with both CDs induces the expression of the lysosome-associated membrane protein 1 (LAMP-1) in NPC patient-derived cells. Remarkably, LAMP-1 overexpression in HeLa cells rescued U18666A-induced cholesterol accumulation suggesting a role of LAMP-1 in cholesterol trafficking. We propose that HPβCD and HPγCD facilitate cholesterol export from the LE/LY compartments via the LAMP-1 protein, which may play a crucial role in cholesterol trafficking at the LE/LY compartments when there is no functional NPC1 protein. Together, this study uncovers new cellular mechanisms for cholesterol trafficking, which will contribute to development of novel therapeutic approaches for lysosomal storage diseases.

## Introduction

Lysosomes are acidic, membrane-bound organelles that play a crucial role in cholesterol metabolism. The majority of cellular demand for cholesterol is made through the receptor-mediated endocytosis of low-density lipoprotein (LDL) from plasma. Following an entry into cells, LDL is transported to the lysosomes via the endosomal compartments. An acid lipase in the lumen of the lysosome hydrolyzes cholesteryl esters in LDL^1^, and the free cholesterol then exits the lysosomal compartment in order to reach other cellular compartments such as the plasma membrane and the endoplasmic reticulum (ER) where it performs both structural and regulatory roles^2–4^. A very little is known about how cholesterol is trafficked from lysosomes or late endosomes to other cellular organelles. Because late endosomes (LE) and lysosomes (LY) share many properties, we will henceforth refer to them indistinctively as either lysosomes or late endosomes/lysosomes (LE/LY).

Niemann–Pick type C (NPC) disease is a fatal hereditary disorder characterized by neurodegeneration, hepatosplenomegaly, and the accumulation of cholesterol and other lipids in lysosomes, and has implicated two lysosomal proteins NPC1 and NPC2 for the process of cholesterol exit from the LE/LY compartments^5^. Both proteins have cholesterol-binding property and homozygous mutations in either NPC1 or NPC2 cause lysosomal lipid accumulation and NPC disease, suggesting an essential role of these proteins in cholesterol export from the LE/LY compartments^6^.

Intracellular level and distribution of cholesterol is tightly regulated by receptor-mediated endocytosis of LDL, de novo biosynthesis, and intracellular trafficking between multiple organelles. Majority of the studies have focused on cholesterol uptake and biosynthesis; however, mechanisms of cholesterol trafficking towards the plasma membrane, plasma membrane to the ER via lysosomes, ER to the plasma membrane via lysosomes, and towards mitochondria are not well understood. Cholesterol levels in ER regulate de novo biosynthesis and uptake of cholesterol by controlling the proteolytic activation of the sterol regulatory element-binding proteins and the re-esterification of cholesterol by the ER-resident acyl CoA:cholesterol acyltransferase. For use and storage at other cellular compartments, cholesterol has to exit the lysosomes. A model has been proposed for cholesterol trafficking at the LE/LY compartments, in which the NPC2 protein binds cholesterol in the lysosomal lumen and then transfer cholesterol to the N-terminal domain of the lysosomal membrane-spanning NPC1 protein^5^. Cholesterol is then transported out of the LE/LY compartments by some unknown mechanisms. Cholesterol trafficking at the LE/LY compartments has gained interest recently due to its significance in understanding the mechanisms of NPC disease as well as other lysosomal storage disorders.

Cyclodextrins (CDs), a family of cyclic oligosaccharides consisting of α-d-glucopyranose molecules joined by alpha-1-4-glycosidic linkages, can form complexes with a number of poorly water-soluble molecules including cholesterol through their hydrophobic cavity and enhance the solubility of the guest molecules via their hydrophilic outer surface^7^. Because of this cholesterol-binding property, selected CD derivatives have been investigated as a potential therapy for NPC disease. One such CD, 2-hydroxypropyl-β-cyclodextrin (HPβCD), has been shown to alleviate the cholesterol accumulation defect in *NPC1* mutant cells^8^. In preclinical studies, a direct brain injection of HPβCD into an NPC mouse model slowed the progression of neuronal loss and improved its survival^9–11^. Similar results were observed in a feline model of NPC disease, with an intra-cisternal administration conferring attenuated neurodegeneration and prolonged survival^12^. More recently, an intrathecal cyclodextrin injection in a single-NPC1 patient led to an increase in cholesterol redistribution in the central nervous system and improved vertical gaze palsy, a clinical indicator of NPC-linked neurodegeneration^13^. In 2013, due to the encouraging preclinical data, the HPβCD entered into first-in-human clinical trials at the NIH Clinical Center. The HPβCD successfully completed phase 1/2a clinical trial designed to test its safety and effectiveness. The study showed that intrathecal injections of HPβCD decreased the progression of neurological disease in NPC patients^14^. Based on the promising data of phase 1–2 clinical trial, currently, the HPβCD is being tested in a multicenter, multinational phase 2b/3 clinical trial (NCT02534844). Although the efficacy of HPβCD in NPC disease is well established, the mechanism is still unknown. A recent study demonstrated that 2-hydroxypropyl-γ-cyclodextrin (HPγCD) can reduce cholesterol accumulation in the NPC patient-derived cells and prolong survival in NPC model mice^15^. One study also showed that another CD derivative, methyl-β-cyclodextrin (MβCD), binds and activates AMP-activated protein kinase (AMPK), which was essential for MβCD-mediated reduction of cholesterol storage in NPC1 mutant cells^16^. This study provided the evidence of direct binding of MβCD to the molecular target and reducing the cholesterol levels in NPC1 mutant cells. It is important to identify the molecular target of HPβCD and HPγCD in order to develop drugs for NPC. We recently screened twelve derivatives of α, β, and γ CDs with different functional substitutions for their ability to modulate intracellular cholesterol accumulation; of the 12 CD derivatives tested, HPβCD and HPγCD were able to rescue the cholesterol accumulation defect in NPC patient-derived cells^17^. However, how HPβCD or HPγCD triggers the cholesterol egress from the LE/LY compartments and normalize cellular cholesterol homeostasis is unanswered. Understanding these mechanisms will improve the cyclodextrin-based therapy and may reveal new drug targets for NPC disease. Therefore, it is important to understand the mechanisms by which the CDs rescue the intracellular cholesterol accumulation defect and to determine the cellular proteins or pathways that are critical for modulating intracellular cholesterol homeostasis.

In the current studies, we used a proteomic approach to elucidate the mechanism of cyclodextrin-mediated exit of cholesterol from lysosomes. We identified a number of candidate proteins differentially expressed in *NPC1* mutant cells compared to the wild-type cells; interestingly, HPβCD and HPγCD were shown to modulate expression levels of most of these proteins. Through overexpression and knockdown approaches, we determined that the lysosome-associated membrane protein 1 (LAMP-1) mediates the cyclodextrin-induced exit of cholesterol from lysosomes when there is no functional NPC1 protein. We use these data to develop a hypothetical working model for cholesterol trafficking at the LE/LY compartments in which the lysosomal membrane protein LAMP-1 substitutes the NPC1 protein and accepts cholesterol from the lysosomal luminal protein NPC2, prior to transferring cholesterol to other cellular compartments such as the ER membrane or plasma membrane.

## Results

### Effect of CDs on cell viability in fibroblasts derived from NPC patient and healthy donor

**Fig. 1:**
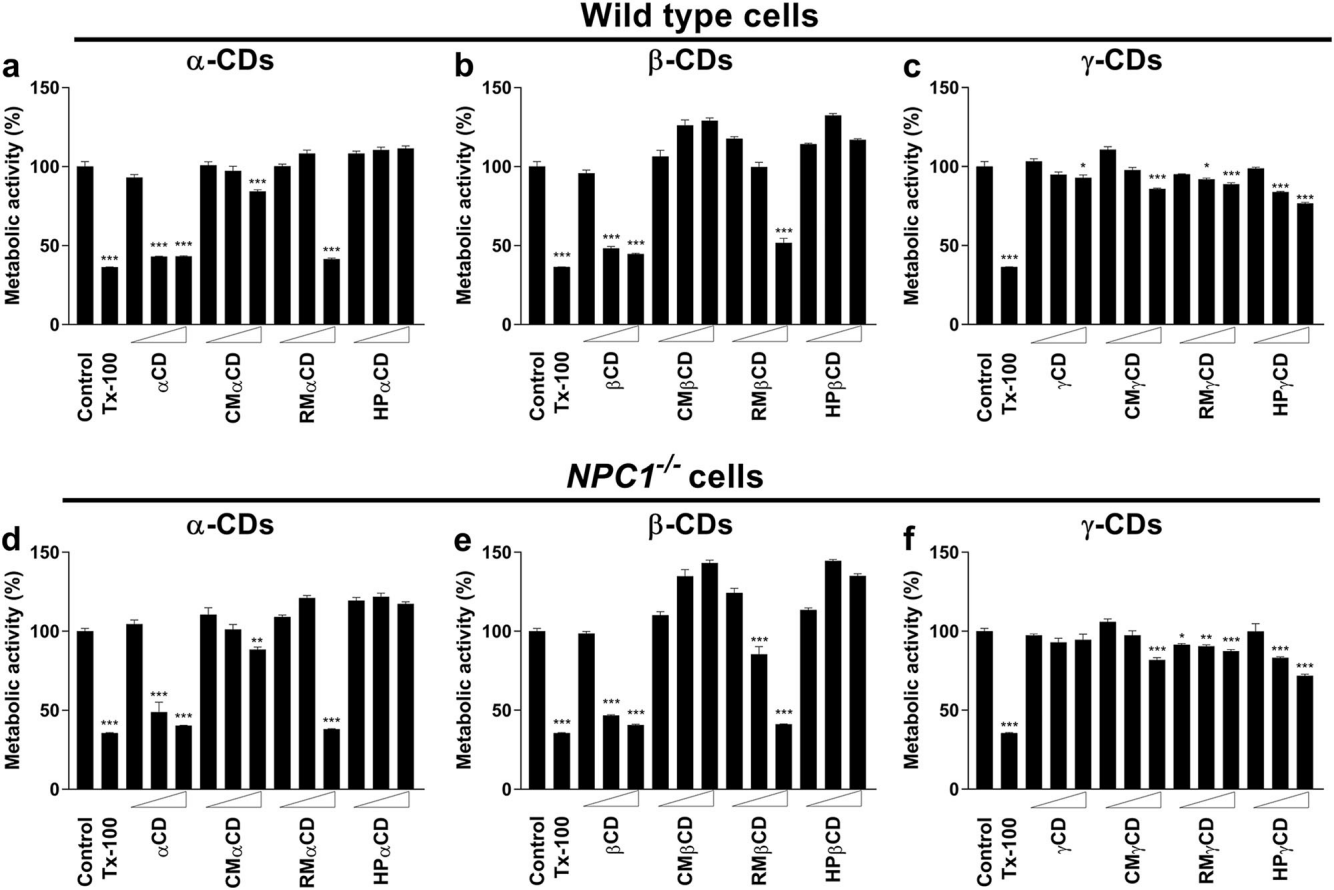
**Effect of CD derivatives on cell viability.** The wild-type (healthy donor) cells (**a**, **b**, **c**) or *NPC1*^*−/−*^ cells (**d**, **e**, **f**) were treated with 1, 5, or 10 mM of CD derivatives for 24 h. Metabolic activity was measured by MTS assay and expressed as a percentage of metabolic activity (**a**–**f**). There was no significant difference in MTS activity at 1 mM concentration in all of the CD derivatives. However, native or RM-modified CDs (except γ-CDs) showed decreased metabolic activity at 5 or 10 mM concentration in both cells. CM- and HP-modification abrogated CD-induced decrease in metabolic activity. Triton X-100 (0.1% v/v) was used as a positive control. Data are mean ± S.E.M. of quadruplicates and a representative of three independent experiments. Symbols indicate the relative level of significance compared with control (*P < 0.01; **P < 0.001; ***P < 0.0001)

First, we tested the effect of various CD derivatives on cell viability (or metabolic activity) in human fibroblasts from a healthy donor or from a NPC patient with a defect in *NPC1* gene. The CDs tested include the native forms of α-, β-, and ɣ-CDs as well as their chemical modifications with hydroxypropyl (HP), randomly methylated (RM), or carboxymethyl (CM) group. Figure 1 shows that 1 mM concentration of all of the CD derivatives did not change cell viability (as depicted by metabolic activity). However, at 5 or 10 mM concentration, native or RM-modified α- and β-CD treatment significantly decreased the metabolic activity in both cell types (Fig. 1a, b, d, e), but γ-CDs did not affect cell viability (Fig. 1c, f). Interestingly, substitutions with carboxymethyl or hydroxypropyl group in α-CDs and β-CDs significantly abrogated their toxicity, as CM-α-CD and CM-β-CD did not alter metabolic activity at 5 mM or 10 mM concentration in both cell types. Modification of α-, β- or γ-CD with hydroxypropyl group did not show any toxicity in both cell types except that only slightly decreased metabolic activity was observed in cells treated with HP-γ-CD at 5 or 10 mM concentration. Modification of α- or β-CD with methyl group did not show toxicity at 5 mM concentration, but methylated α-CD and β-CD significantly decreased metabolic activity at 10 mM concentration. Modification of γ-CD with methyl group slightly decreased metabolic activity at 5 or 10 mM in both cell types. Together, these data suggest that neither native CDs nor any CD derivatives are toxic at 1 mM concentration in healthy as well as *NPC1*^*−/−*^ fibroblasts and therefore, we selected this concentration for our study.

### Effects of CDs on lysosomal accumulation of cholesterol

**Fig. 2:**
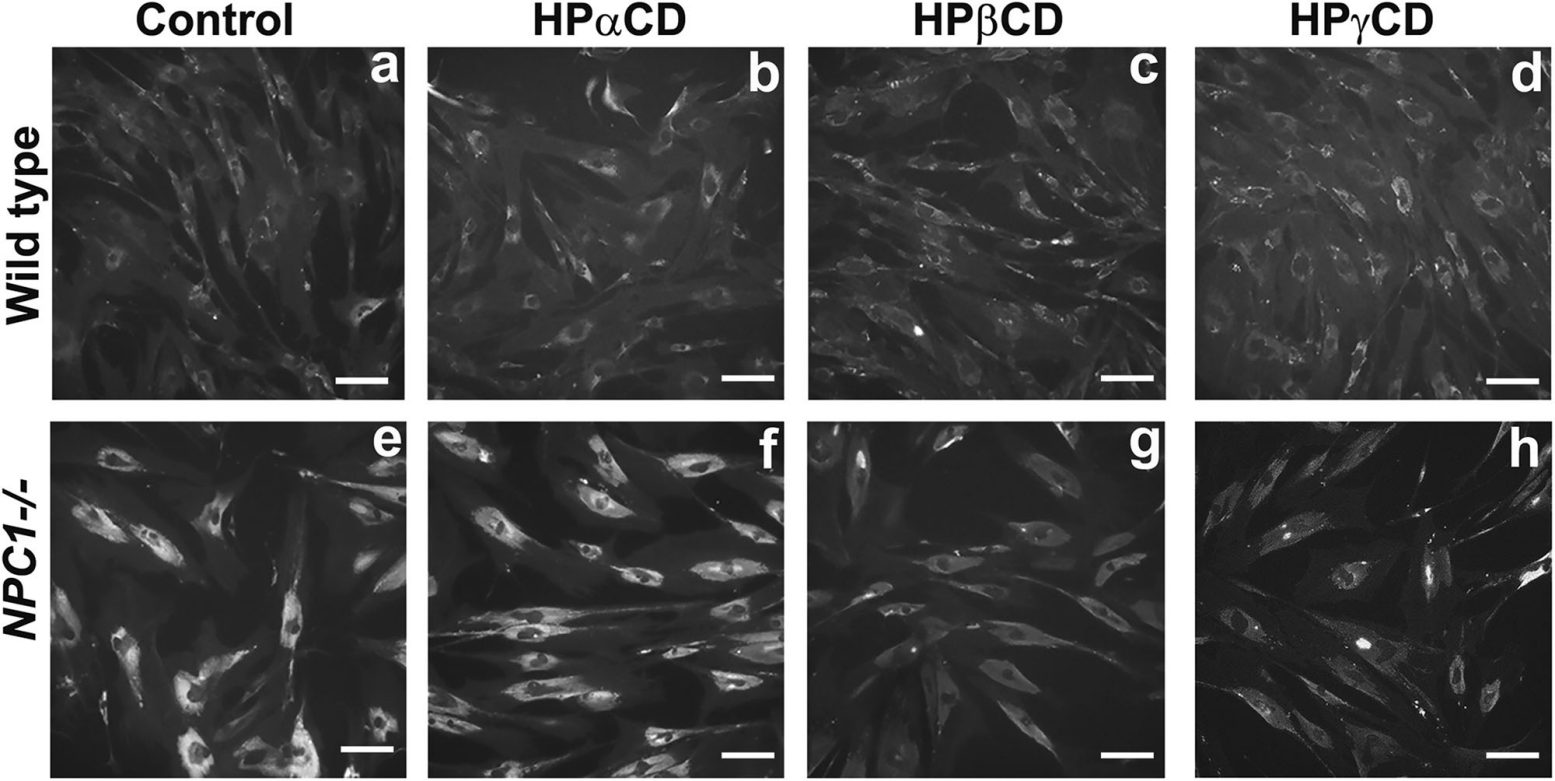
**Effect of CD derivatives on cholesterol accumulation in*NPC1***^***−/−***^ **cells.** Primary fibroblast cells from a healthy donor or NPC patient were incubated with CD derivatives (1 mM) for 72 h and the levels of free cholesterol in cells were determined by staining with Filipin. Data shown are a representative of three independent experiments. Wild-type, primary fibroblast cells from a healthy donor; *NPC1*^*-/-*^ cells, primary fibroblast cells from an NPC patient. Scale bar = 50 μm

We examined the effects of the CD derivatives on the levels of free cholesterol in lysosomes in *NPC1* mutant fibroblast cells using filipin, a fluorescent compound that specifically binds to unesterified cholesterol present in lysosomes. As depicted in microscope image, *NPC1* mutant cells (Fig. 2e) showed a robust accumulation of cholesterol as compared to healthy skin fibroblast cells (Fig. 2a) reflecting a defect in cholesterol exit from lysosomes. Interestingly, HPβCD or HPγCD treatment remarkably reduced cholesterol levels in *NPC1* mutant cells (Figure 2g, h) whereas HPαCD did not affect the intracellular cholesterol accumulation (Figure 2f). Therefore, we have selected HPβCD and HPγCD to further investigate the molecular mechanisms by which these CDs facilitate cholesterol exit from lysosomes and reduce the cholesterol levels in *NPC1* mutant cells.

### Effects of HPβCD and HPγCD treatments on the expression of cellular proteins

**Fig. 3:**
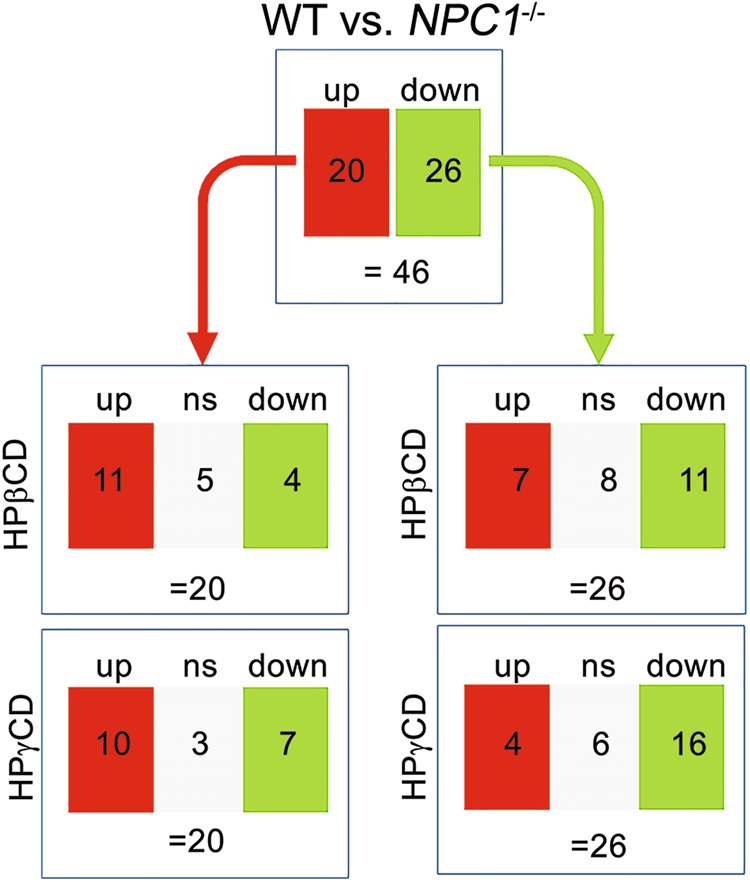
**Neimann–Pick disease type C 1-specific and cyclodextrin-regulated proteins identified from a mass-spectrometric platform.** LC-MS proteomic analysis of NPC patient-derived fibroblasts that were treated with HPβCD or HPγCD. Cell lysates were prepared from NPC patient-derived fibroblasts or primary fibroblast cells from a healthy donor after treatment with HPβCD or HPγCD (1 mM, 72 h). Protein samples were subjected to LC-MS analysis (MudPIT) and peptide identification using PEAKS8.0 and MyriMatch. Untreated fibroblasts from an NPC patient and untreated fibroblasts from a healthy donor were included as controls. Four groups of samples were analyzed; untreated healthy donor cells (Healthy), untreated NPC patient cells (NPC), NPC patient cells treated with HPβCD (NPC/HPβCD), and NPC patient cells treated with HPγCD (NPC/HPγCD). The flow chart shows a total 46 differentially expressed proteins in *NPC1*^*-/-*^ cells compared to that of cells from a healthy donor (top). HPβCD (middle) or HPγCD (bottom) treatment modulated expression levels of these proteins. A pairwise comparison was performed between WT vs. NPC; WT vs. NPC + HPβCD; WT vs. NPC + HPγCD

To understand the potential mechanisms of HPβCD- or HPγCD-mediated modulation in intracellular cholesterol homeostasis, we attempted to identify differentially expressed proteins involved in cholesterol metabolism and trafficking. We utilized automated multidimensional protein identification technology (MudPit), which is a shotgun proteomics approach usually applied to obtain overviews of protein expression in biological samples^18^. We applied standard label-free quantification of high mass resolution LC-MS data^19^ for high throughput analysis of protein expression in NPC1 mutant cells. LC-MS/MudPIT analysis showed a robust change in protein expression profile in NPC patient-derived cells compared to cells derived from a healthy donor, which is modulated remarkably, when NPC patient-derived cells were subjected to HPβCD or HPγCD treatment. First, we compared protein levels between healthy donor-derived cells (the wild-type control) and NPC patient-derived cells (*NPC1* mutant) and identified 46 differentially expressed proteins (annotated by protein database) whose levels differed more than twofold in *NPC1* mutant cells compared to wild-type cells (Table 1). These proteins may potentially be implicated in the control of cholesterol homeostasis in normal cells with abnormal expression of these proteins resulting in the cholesterol trafficking defect in *NPC1* mutant cells (Fig. 3 and Table 1). The modulation of these proteins might be essential to correct the cholesterol trafficking defect in *NPC1* mutant cells. Remarkably, 35 proteins out of the these 46 proteins were altered more than twofold in *NPC1* mutant cells upon treatment with HPβCD or HPγCD, with their levels being shifted toward those of healthy donor cells from those of NPC patient cells (Fig. 3). These 35 proteins include: 12 proteins upregulated by both HPβCD and HPγCD, 5 proteins upregulated by HPβCD only, 1 protein upregulated by HPγCD only, 10 proteins downregulated by both HPβCD and HPγCD, 3 proteins downregulated by HPβCD only, and 4 proteins downregulated by HPγCD only. We hypothesized that some of these 35 proteins may play a critical function in CD-mediated modulation of the intracellular cholesterol trafficking. Though, the MudPIT technique is fast and sensitive with good reproducibility, it lacks the ability to provide quantitative information and relies on label-free quantification method^20–22^. Therefore, we validated the quantitative information of our selected proteins with other methods such as western blots. There were 137 or 172 proteins differentially expressed in *NPC1* mutant cells upon treatment with HPβCD or HPγCD, respectively, when compared to untreated cells (Supplemental Table 1). Interestingly, majority of the differentially expressed proteins were common in HPβCD and HPγCD treated cells. A total 93 proteins were common for HPβCD and HPγCD treatment and 44 proteins were differentially expressed exclusively in HPβCD treated cells and 72 proteins were differentially expressed in HPγCD treated cells (Supplemental Table 1). While we focus on the role of one candidate protein in this study, we can not rule out the involvement of several other proteins in the regulation of cellular cholesterol homeostasis.

**Table 1:** Neimann–Pick disease type C1-specific proteins that are differentially expressed by treatment with HPβCD or HPγCD. LAMP-1 protein was identified as significantly upregulated upon HPγCD treatment (also upon HPβCD treatment when compared with the NPC group) as compared to either healthy or NPC control cells

Accession ID	Protein ID	Name of the protein	*NPC1*^*−/−*^ Con	*NPC1*^*−/−*^ + HPβCD	*NPC1*^*−/−*^ *+* HPγCD
NP_001276332	MMS19	MMS19 homolog, cytosolic iron–sulfur assembly component	9.35	176.17	3.92
XP_005246524	GLS	Glutaminase	0.2	2.08	0.47
NP_001340	DARS	Aspartyl-tRNA synthetase	115.28	43.37	185.52
XP_011529432	FLNA	Filamin A	0.43	0.24	0.07
NP_005336	HSPA1A	Heat shock protein family A (Hsp70) member 1A	4.01	0.98	14.89
NP_002878	RARS	Arginyl-tRNA synthetase	0.04	1.61	3.26
NP_001030168	RPL14	Ribosomal protein L14	5.59	0.18	0.01
XP_005267356	AHNAK2	AHNAK nucleoprotein 2	0.36	0.38	0.07
NP_004795	CIAO1	Cytosolic iron–sulfur assembly component 1	0.03	0.38	1.08
NP_001814	CKB	Creatine kinase B	2.15	2.51	0.13
XP_011533095	GOLGA3	Golgin A3	0.05	1.72	3.89
NP_000975	RPL23A	Ribosomal protein L23a	0.47	0.85	0.07
NP_006504	SARS	Seryl-tRNA synthetase	2.59	3.42	0.42
NP_037466	GMPPB	GDP-mannose pyrophosphorylase B	0.03	0.04	0.97
NP_002619	PFN2	Profilin 2	0.31	0.25	0.05
NP_001287921	EIF3K	Eukaryotic translation initiation factor 3 subunit K	7.26	3.65	0.34
NP_006657	RUVBL2	RuvB like AAA ATPase 2	7.17	1.38	0.34
NP_001011	RPS16	Ribosomal protein S16	0.1	0.27	0.6
NP_000980	RPL30	Ribosomal protein L30	13.34	3.56	9.95
NP_001687	ATP6V1E1	ATPase H + transporting V1 subunit E1	0.5	0.43	0.02
NP_001419	ENO1	Enolase 1	0.05	1.29	0.27
NP_009057	VCP	Valosin-containing protein	2.51	2.35	4.89
NP_002583	PCNA	Proliferating cell nuclear antigen	0.5	8.3	0.08
NP_579899	MYOF	Myoferlin	34.77	10.59	4.08
NP_006127	CAPZA2	Capping actin protein of muscle Z-line alpha subunit 2	0.21	0.11	0.22
NP_001018083	PCK2	Phosphoenolpyruvate carboxykinase 2, mitochondrial	61.89	19.43	3.63
NP_001279	CLIC1	Chloride intracellular channel 1	0.03	5.21	2.19
NP_067072	SNX6	Sorting nexin 6	0.03	2.06	0.04
NP_066977	PSAT1	Phosphoserine aminotransferase 1	0.03	3.64	0.31
NP_001075109	PRKDC	Protein kinase, DNA-activated, catalytic polypeptide	0.26	0.07	0.01
NP_000393	G6PD	Glucose-6-phosphate dehydrogenase	0.01	2.15	0.84
NP_006182	PA2G4	Proliferation-associated 2G4	3.66	0.2	1.74
XP_005253351	STRAP	Serine/threonine kinase receptor associated protein	0.16	2.51	0.68
NP_001257356	PSMD5	Proteasome 26S subunit, non-ATPase 5	0.35	0.77	0.02
NP_055416	EHD2	EH domain containing 2	3.87	0.1	0.25
NP_056991	LAP3	Leucine aminopeptidase 3	2.25	1.28	6.33
NP_001185709	ARPC4	Actin related protein 2/3 complex subunit 4	2	0.08	0.78
NP_001257411	PSMD11	Proteasome 26S subunit, non-ATPase 11	0.07	0.82	0.01
XP_011523798	NPEPPS	Aminopeptidase puromycin sensitive	0.4	0.01	0.03
NP_001137290	SERPINB2	Serpin family B member 2	16.62	25.23	8.1
NP_620164	CMBL	Carboxymethylenebutenolidase homolog	0.43	0.01	0.02
NP_001121188	ETFA	Electron transfer flavoprotein alpha subunit	2.05	11.03	3.67
NP_004981	MARS	Methionyl-tRNA synthetase	8.58	1.22	0.3
NP_002787	PSMB4	Proteasome subunit beta 4	78.05	21.33	9.59
NP_003133	SSB	Sjogren syndrome antigen B	0.15	1.19	0.17
NP_005552	LAMP1	Lysosomal associated membrane protein 1	0.43	1.69	3.71

### LAMP-1 expression and lysosome distribution in *NPC1* mutant cells

**Fig. 4:**
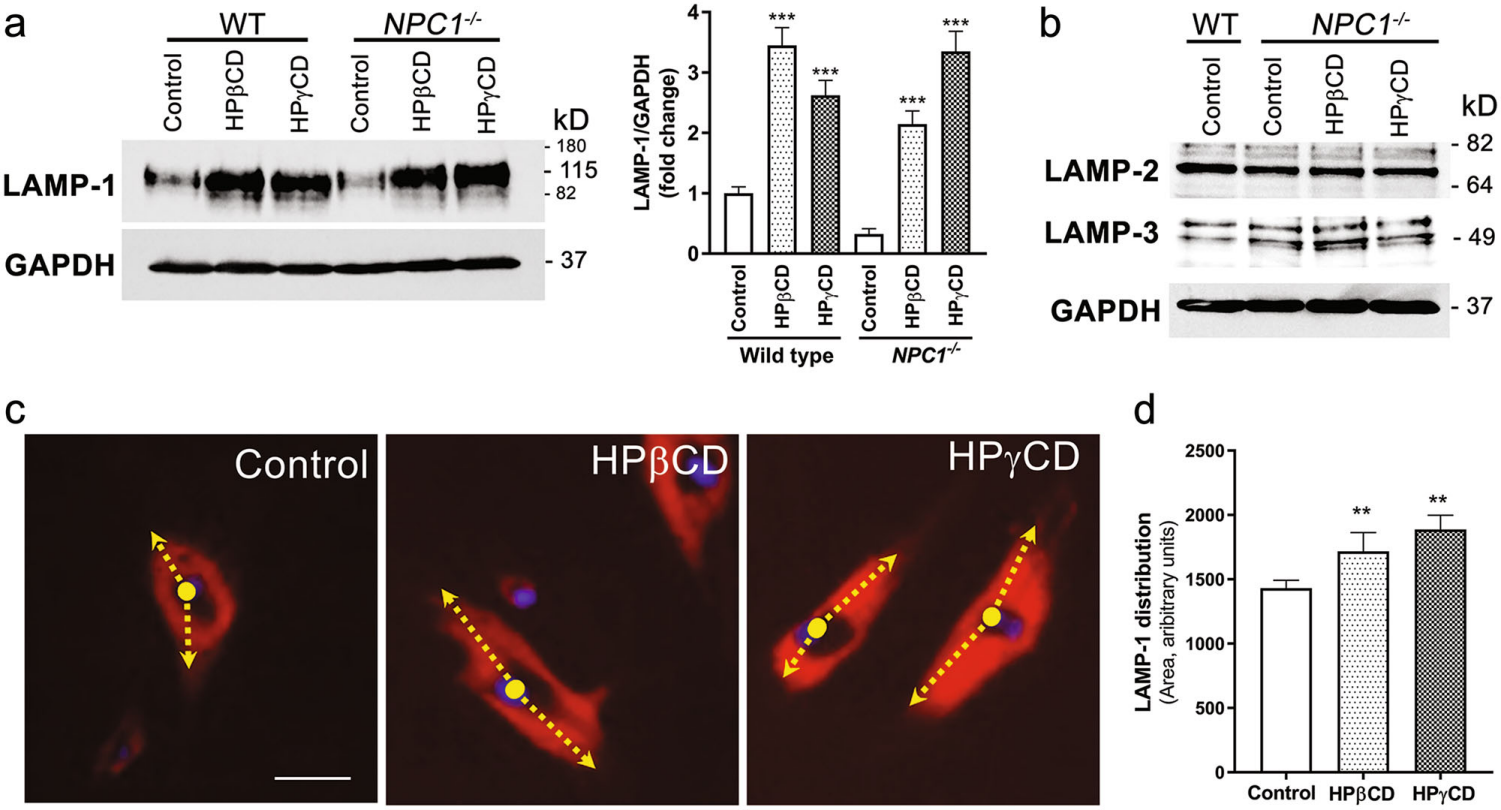
**HPβCD or HPγCD treatment induces LAMP-1 expression and causes a change in lysosomal positioning in*NPC1*** **mutant cells.** The healthy (wild-type) cells or *NPC1*^*−/−*^ cells treated with HPβCD or HPγCD for 72 h were lysed for immunoblotting (**a**, **b**) or stained for LAMP-1 (**c**). **a** The representative western blot and bar diagram of three experiments show that wild-type or *NPC1*^*−/−*^ cells treated with either HPβCD or HPγCD showed significantly higher levels of LAMP-1 expression (*p* < 0.001). The error bars shows mean ± S.E.M. of fold change calculated by densitometry analysis. **b** The representative western blot of three experiments shows that neither HPβCD nor HPγCD treatment changed expression of LAMP-2 or LAMP-3 in wild-type or *NPC1*^*−/−*^ cells. **c** Immunostaining micrographs show LAMP-1 (red, a lysosome marker) and DAPI (blue, a nucleus marker) staining. The arrow represents the distribution of LAMP-1 from the center of the nuclei. Data depict that the LAMP-1 protein is mostly confined to the area near the nuclear envelop in control *NPC1*^*−/−*^ cells, whereas it is distributed more widely throughout the cytoplasm when cells were treated with either HPβCD or HPγCD. Images are a representative of at least three random fields of three experimental replicates. Scale bar = 100 μm. **d** The LAMP-1 distribution per cell was quantified by measuring the area of fluorescence. Data are mean ± S.E.M. and representative of three experiments

Out of 35 candidate proteins differentially expressed in *NPC1* mutant cells treated with HPβCD or HPγCD, we selected the LAMP-1 protein to further investigate its role in cholesterol trafficking at the LE/LY compartments. According to MudPit data, the LAMP-1 protein was upregulated by HPβCD and HPγCD in *NPC1* mutant cells. Therefore, we verified this finding by Western blotting (Fig. 4a), which shows that the lysosomal membrane protein LAMP-1 was upregulated by HPβCD and HPγCD consistent with the MudPit data. However, LAMP-2 and LAMP-3 did not show any change in expression in *NPC1* mutant cells after HPβCD or HPγCD treatment (Fig. 4b).

As LAMP-1 is a marker of lysosomes, we determined whether lysosomal positioning is altered upon treatment with HPβCD or HPγCD. Interestingly, lysosomes were distributed more widely within the cell upon HPβCD or HPγCD treatment in *NPC1* mutant cells whereas the lysosomes were clustered near the cell center in untreated *NPC1* mutant cells (Fig. 4c, d). These results are consistent with previous studies demonstrating that cholesterol accumulation or NPC1 deficiency is associated with a defect in LE/LY mobility^23–25^, and suggest that cholesterol trafficking at the LE/LY compartments may be important for maintaining the biogenesis, distribution, and function of lysosomes.

### Role of LAMP-1 in cholesterol exit from lysosomes

**Fig. 5:**
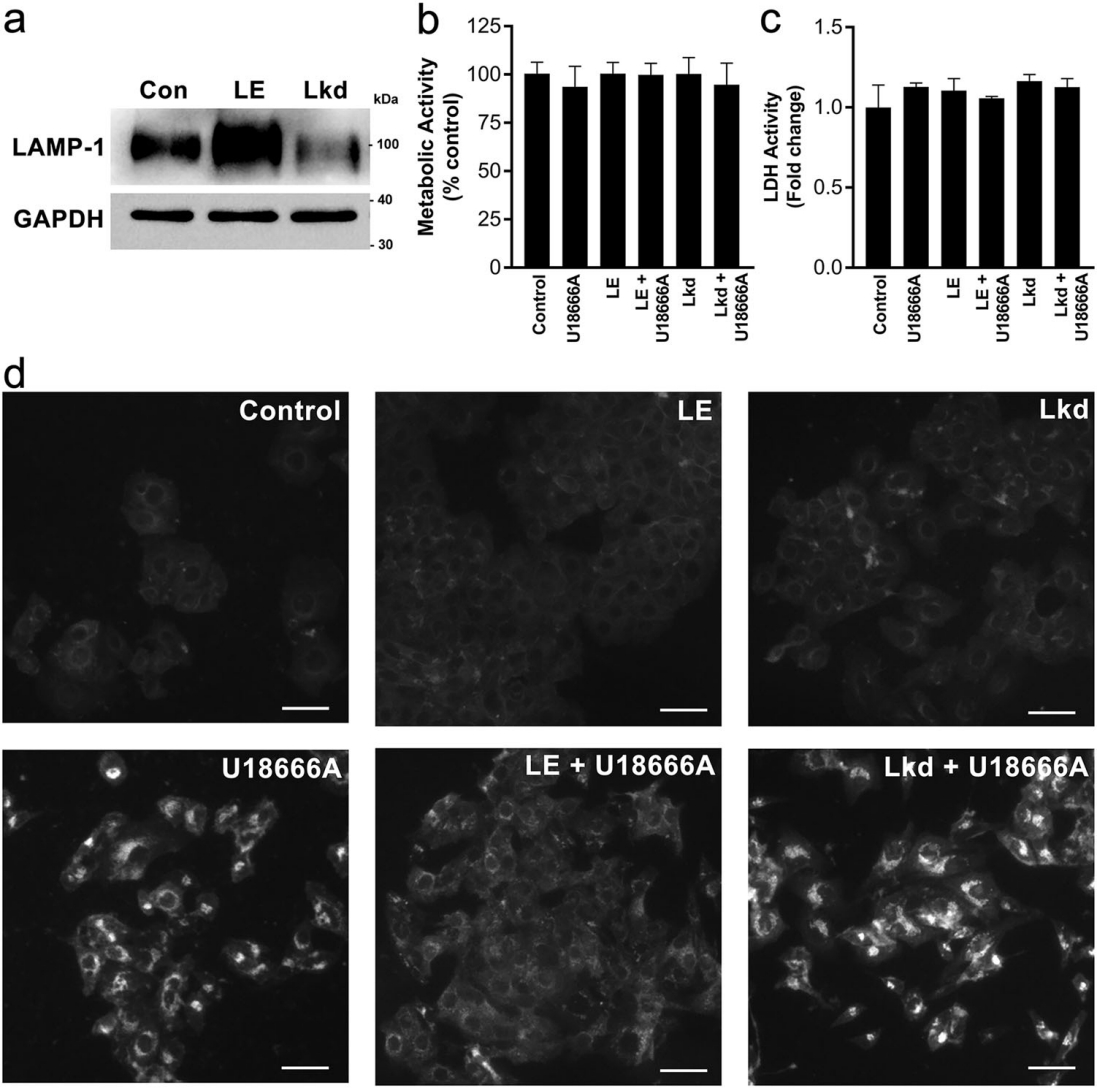
**LAMP-1 is essential for cyclodextrin-mediated cholesterol export from lysosomes.** Verification of HeLa cells either overexpressing LAMP-1 (HeLa-LE) or stably carrying shRNA for LAMP-1 (Hela LAMP-1 knockdown or HeLa-kd) by western blot (**a**). HeLa-LE shows significantly increased levels of LAMP-1, while HeLa-Lkd shows decreased levels of LAMP-1 compared to the HeLa cells transfected with a control plasmid. Control or transfected HeLa cells were treated with U18666A (5 μg/ml) for 48–72 h, and metabolic activity (**b**) and LDH activity (**c**) were measured. Data are mean ± S.E.M. and representative of three experiments. None of transfected cell lines showed toxicity after three days of treatment with U18666A. HeLa cells lines were analyzed for cholesterol accumulation by filipin imaging (**d**). U18666A-induced cholesterol accumulation in control cells; however, LAMP-1 overexpression (HeLa-LE) suppressed U18666A-induced cholesterol accumulation. LAMP-1 knockdown (HeLa-Lkd) did not show any protection against cholesterol accumulation and showed higher accumulation of cholesterol compared to control. Microscope images are a representative of at least three random fields of three experimental replicates. Scale bar = 50 μm

To understand whether the LAMP-1 protein has any role in CD-mediated cholesterol exit from lysosomes in *NPC1* mutant cells, we made two stable cell lines of HeLa using a lentivirus-based plasmid vector system. HeLa cells either overexpressing the LAMP-1 protein (Hela-LE) or stably carrying shRNA specific for LAMP-1 (Hela LAMP-1 knockdown or HeLa-Lkd) showed significantly increased or decreased levels of LAMP-1, respectively, as compared to cells transfected with a negative control plasmid (Fig. 5a). Both HeLa-LE and HeLa-Lkd cell lines did not show any sign of cytotoxicity due to overexpression or knockdown of LAMP-1 (Fig. 5b, c). We used U18666A, an inhibitor of cellular cholesterol trafficking that induces an *NPC1* mutation phenotype, to investigate the role of the LAMP-1 protein in cholesterol trafficking at the LE/LY compartments. Treatment with U18666A (5 μg/ml for 3 days) did not alter the metabolic activity of any of the HeLa cell lines (Fig. 5b, c). Filipin imaging showed that U18666A treatment significantly increased cholesterol accumulation in control HeLa cells (Fig. 5d, left). Interestingly, LAMP-1 overexpression rescued the cholesterol accumulation defect in HeLa cells (Fig. 5d, middle) whereas LAMP-1 knockdown did not alleviate the cholesterol accumulation defect (Fig. 5d, right). Moreover, LAMP-1 knockdown caused a slight increase in the levels of cholesterol compared to the control cells upon treatment with U18666A. Taken together, these data suggest that the LAMP-1 protein plays an important role in cholesterol trafficking at the LE/LY compartments, and potentially re-positions the lysosomes toward other organelles and/or the plasma membrane in order to maintain cholesterol homeostasis.

## Discussion

Herein, we have demonstrated that HPβCD and HPγCD upregulate the expression of the lysosomal membrane protein LAMP-1 and facilitate cholesterol trafficking at the LE/LY compartments, rescuing the cholesterol accumulation defect in NPC patient-derived fibroblast cells (*NPC1* mutant). By using a proteomic approach, we identified a number of proteins that may potentially be implicated in cholesterol metabolism or trafficking. It is very well established that HPβCD (and possibly HPγCD) modulates intracellular cholesterol accumulation and alleviates NPC disease^5,10,15,26,27^. However, the molecular mechanisms by which HPβCD or HPγCD facilitates the exit of cholesterol from lysosomes and reduces the intracellular cholesterol accumulation in *NPC1* mutant cells are largely unknown. Our data showed that both HPβCD and HPγCD rescue the cholesterol accumulation defect in *NPC1* mutant cells (Fig. 2), despite the differences in their physicochemical properties^17^. While HPγCD has a much weaker capacity to solubilize or extract cholesterol compared to HPβCD^17,28^, HPγCD was very effective in alleviating the cholesterol accumulation defect in a cell culture model or mouse model of NPC disease^15,17^. The view that the CDs alleviate the cholesterol accumulation defect in NPC simply by binding and/or extracting cholesterol should be re-evaluated. Together, these findings suggest that CD-mediated modulation of intracellular cholesterol accumulation may involve a multitude of mechanisms^17^. We demonstrated that treatment with HPβCD or HPγCD induces changes in the expression of a number of proteins (Table 1) involved in diverse cellular pathways such as intracellular vesicle transport, lipid metabolism, and cell growth, etc. Further study is warranted to better understand the functional significance of the cellular signaling pathways and proteins that are induced by HPβCD and HPγCD.

The lysosomal membrane glycoproteins LAMP-1 and LAMP-2 are the two most abundant proteins in the lysosome lining the limiting membrane^29^. The LAMP-1 and LAMP-2 proteins are 37% identical and comprised of a short cytoplasmic domain, a single transmembrane domain, and a highly, N- and O-glycosylated luminal domain, and may have overlapping functions^30–32^. However, relatively little is known about the functions of these proteins in cholesterol homeostasis and they are often presumed to be structural components. One study showed that LAMPs are required for fusion of lysosomes with phagosomes^33^. Recently, the role of LAMPs in cholesterol homeostasis has gained interest. LAMP-2-deficient cells showed cholesterol accumulation, which was corrected by LAMP-2 expression^34,35^. A proteomic study identified LAMP-1 and LAMP-2 as potential cholesterol-binding proteins^36^. It has been suggested that the LAMP-1/-2 proteins can bind cholesterol directly and perhaps facilitate cholesterol export from lysosomes^37^. Despite these clues, the detailed functions of LAMP-1 in cholesterol trafficking have remained unclear.

In this study, we demonstrated that LAMP-1 overexpression can rescue pharmacologically (U18666A) induced cholesterol accumulation in HeLa cells, a mimic of *NPC1 or NPC2* mutation. However, LAMP-1 knockdown did not induce cholesterol accumulation in untreated HeLa cells, which have functional NPC1 and NPC2 proteins. These results suggest that there is a functional redundancy between LAMP-1 and NPC1/NPC2 proteins for the regulation of cholesterol homeostasis. Based on these data, we speculate that in some circumstances, especially in the absence of a functional NPC1 protein, LAMP-1 is capable of binding cholesterol and directing it to the lysosomal membrane for trafficking to the plasma membrane and/or the ER. If this hypothesis is true, then *NPC1* mutant cells should have higher LAMP-1 expression levels than the control wild-type cells to maintain intracellular cholesterol homeostasis. In contrast, we observed the opposite results; *NPC1* mutant cells showed lower levels of LAMP-1 compared to the wild-type control cells. Considering that our observation is based on only one cell line, further investigations are warranted (including more cell lines) to confirm this possibility. It seems that an alteration in the levels of intracellular cholesterol does not directly provide a signal for the cellular machinery to upregulate LAMP-1 expression in order to maintain cholesterol homeostasis in *NPC1* mutant cells. In this scenario, treatment with cyclodextrins (HPβCD and HPγCD) has more significance for inducing LAMP-1 expression in *NPC1* mutant cells (which maintain low levels of LAMP-1 otherwise) to restore cholesterol homeostasis. Therefore, this study found a new role of LAMP-1 in intracellular cholesterol homeostasis as well as the potential for the cyclodextrin-based therapeutic strategies for NPC disease.

One of the major unanswered questions in cellular cholesterol homeostasis is how free cholesterol exits the LE/LY compartments and reaches other cellular compartments such as the ER and the plasma membrane. It was suggested that the lysosomal luminal protein NPC2 binds cholesterol released from LDL and hands-off to the N-terminal domain of the lysosomal membrane-spanning protein NPC1, which then inserts the cholesterol into the lysosomal membrane^5^. Crystallography, genetic and in vitro binding studies suggested that NPC2 and NPC1 bind to cholesterol in opposite orientation such that NPC2 exposes the 3β-OH group and buries the isooctyl side chain deep within a hydrophobic pocket; however, when NPC1 binds cholesterol, it exposes the isooctyl side chain and buries the 3β-OH group^38,39^. This change in the orientation of cholesterol during its transfer from NPC2 to NPC1 appears to allow NPC1 to insert the isooctyl side chain of cholesterol into the lysosomal membrane prior to trafficking to other cellular compartments. Because LAMP-1 is known to bind NPC1, NPC2, and cholesterol^37^, it will be very interesting to study how LAMP-1 is involved in the process of cholesterol exit from the LE/LY compartments. Further studies are necessary to understand whether cholesterol is channeled to LAMP-1 directly or cholesterol is handed-off to LAMP-1 through NPC2 or NPC1 prior to the exit from the LE/LY compartments.

**Scheme 1:**
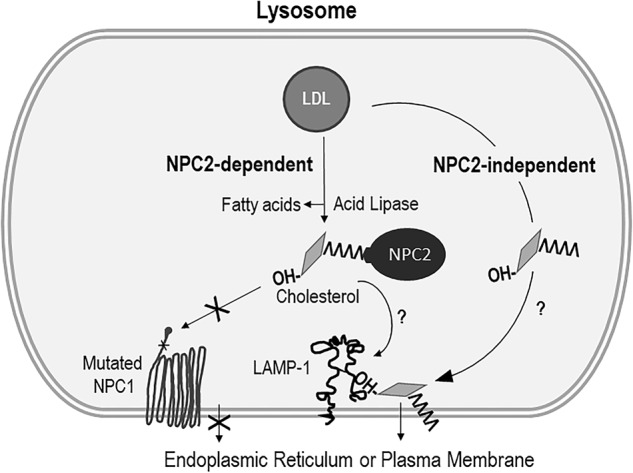
**A hypothetical model of cholesterol egress from lysosome to ER or PM in** ***NPC1*** **mutant cells.** When *NPC1* mutant cells are treated with HPβCD or HPγCD, free cholesterol released from LDL is handed-off to LAMP-1 by NPC2 in an NPC2-dependent manner or it directly bind to LAMP-1

It is possible that the orientation of cholesterol-binding to LAMP-1 is similar to NPC1 rather than to NPC2 (Scheme 1). LAMP-1 is a large lysosomal membrane glycoprotein, whereas NPC2 is a relatively small protein present in the lumen of lysosomes^40^. Moreover, NPC2 alone is not sufficient for cholesterol egress from lysosomes, and NPC1 is essential for this process^6,41^. Our data suggest that the LAMP-1 protein is important in maintaining cholesterol trafficking at the LE/LY compartments, in particular when NPC1 function is absent or limited. Together, these findings support a novel role of LAMP-1 in cyclodextrin-mediated cholesterol homeostasis. However, this hypothesis needs further investigations and will be the focus of our future studies.

## Materials and methods

### Reagents

Cell culture media and reagents were purchased from Thermo Fisher Scientific (Waltham, MA, USA); these include Dulbecco’s modified Eagle’s medium (DMEM), MEM non-essential amino acids, fetal bovine serum (FBS), penicillin, and streptomycin. A CellTiter 96 Aqueous One Solution Cell Proliferation Assay System was purchased from Promega (Madison, WI, USA). An LDH Cytotoxicity Assay Kit was obtained from Thermo Fisher Scientific (Waltham, MA, USA). Filipin III were obtained from Sigma-Aldrich (St. Louis, MO, USA). Cyclodextrins (CDs) were obtained from Cyclolab (Budapest, Hungary). These CDs include αCD, βCD, and γCD of native forms as well as their derivatives with hydroxypropyl (HP), randomly methylated (RM), and carboxymethyl (CM) substitutions. The average degree of substitution (DS) for each CD derivative is as follows: HPαCD, DS = 4.5; HPβCD, DS = 6; HPγCD, DS = 4.5; RMαCD, DS = 11; RMβCD, DS = 12; RMγCD, DS = 12; CMαCD, DS = 3.5; CMβCD, DS = 3.5; CMγCD, DS = 3.5.

### Cell culture and cell lines

Untransformed skin fibroblasts from a healthy control subject (No. GM05659) or skin fibroblasts from a patient with Niemann–Pick disease type C1 (*NPC1*, compound heterozygote for mutations P237S and I1061T, No. GM03123) were purchased from Coriell Institute Cell Repositories (Camden, New Jersey, USA). Cells were grown in a monolayer and maintained in DMEM (with high glucose, l-glutamine, and sodium pyruvate) containing non-essential amino acids, 10% FBS, 100 U/ml of penicillin, and 100 µg/ml of streptomycin at 37 °C in a 5% CO_2_ humidified incubator.

Human embryo kidney cell line HEK293T and human cervical cancer-derived cell line HeLa were obtained from the American Type Culture Collection (Manassas, VA, USA). Both cell lines were grown in a monolayer and maintained in DMEM (with high glucose, l-glutamine, and sodium pyruvate) supplemented with 10% heat-inactivated FBS, 100 U/ml of penicillin, and 100 μg/ml of streptomycin at 37 °C in a 5% CO_2_ humidified incubator.

### Cell viability assay

The effect of CD treatment on cell viability or cytotoxicity were determined using the CellTiter 96 AQueous One Solution Cell Proliferation Assay Kit (Promega, Madison, WI, USA). Briefly, cells were seeded overnight in 96-well plates at a density of 1–3 × 10^4^ cells per well. Cells were treated with a series of dilutions of the CDs. Cells treated with phosphate buffered saline (PBS) and cells treated with Triton X-100 (0.1%) were included as negative and positive controls, respectively. After 24–48 h incubation at 37^o^C, cell viability assay was performed using CellTiter 96 AQueous One Solution Cell Proliferation Assay (Promega), which is based on the conversion of the tetrazolium compound [3-(4,5-dimethylthiazol-2-yl)-5-(3-carboxymethoxyphenyl)-2-(4-sulfophenyl)-2H-tetrazolium, inner salt; MTS] as per the manufacturer’s instructions. The formation of colored formazan product by dehydrogenase enzymes in metabolically active cells was recorded at 490 nm absorbance using xMark microplate absorbance spectrophotometer (Bio-Rad, Hercules, NJ, USA). The data were normalized to the values for untreated control. Cytotoxicity in HeLa cells was performed by Lactate dehydrogenase assay using the LDH Cytotoxicity Assay Kit (Thermo Fisher Scientific, Waltham, MA, USA).

### Measurement of intracellular distribution of cholesterol

Cells were incubated in medium containing CD derivatives at 1 mM for 72 h. Cells were washed three times with PBS and fixed with 3.7% paraformaldehyde for 30 min at room temperature. After washing with PBS three times, cells were stained with Filipin III (12.5 μg/ml in PBS) for 45 min. at room temperature. After washing three times with PBS, cells were mounted in anti-fade mounting medium and images were taken using Nikon fluorescence microscope. The cholesterol accumulation was induced in vitro by treating HeLa cells with U18666A (5 μg/ml; Enzo, Farmingdale, NY, USA) for 48–72 h.

### Proteomics MudPIT analysis

The *NPC1* mutant cells or healthy donor cells treated with HPβCD or HPγCD (1 mM for 72 h) were lysed in the lysis buffer (20 mM Tris-HCl, pH 8.0; 0.1% Triton X-100; 100 µg/ml aprotinin and 100 µM leupeptin protease inhibitors). The cell lysate was centrifuged at 10,000 × *g* for 15 min. Approximately 50 μg total protein was denatured in 8 M urea and 50 mM Tris-HCl, pH 8.0, reduced with 10 mM TCEP for 60 min, alkylated with 50 mM iodoacetamide for 60 min, and then diluted with water to 2 M urea and 50 mM Tris-HCl, pH 8.0. Two micrograms of trypsin (Promega) were added for overnight digestion (18 h), and then the tryptic peptides were desalted using Pierce C18 spin columns (Thermo Fischer Scientific).

Desalted samples were dried in Speed-Vac, resuspended in 5 μl of 0.5% formic acid and loaded onto a 3-phase MudPIT column as described previously^42^. A 10-step MudPIT was executed for LC-MS analysis using an Eksigent™ AS-1 autosampler and an Eksigent™1D Plus nano-LC pump online with an Orbitrap LTQ XL linear ion trap mass spectrometer (Thermo Finnigan) with a nanospray source. MS data acquisition was done in a data-dependent 6-event method (a survey FTMS scan (res. 30,000) followed by five data-dependent IT scans for five consequent most abundant ions). Database searches were done with PEAKS 8.0 and MyriMatch software packs against the forward and reverse human trypsin sequences (as downloaded from GenBank). The parameters for database search were: full tryptic digestion; up to 2 missed cleavage sites; 10 ppm for peptide mass tolerance; 0.5 Da for fragment mass tolerance; cysteine carbamidomethylation (+57 Da) as fixed modification; methionine oxidation (+16 Da) as variable modification. The relative quantification of the identified proteins was performed with the Q module of the PEAKS software pack based on the extracted ion currents of the identified unique peptides’ parent ions.

### Overexpression and gene silencing

HeLa cells in monolayer were transfected with lentiviral construct using jetPrime (Polyplus Transfection, Illkirch, France) according to manufacturer’s guidelines. LAMP-1 expression construct pCMV6-AC-GFP-LAMP-1 (RG219208), LAMP-1 knock down plasmid pGFP-C-shLenti (TL311795), and scrambled shRNA plasmid (TR30021) were purchased from Origene (Rockville, MD, USA). Briefly, two million HeLa cells in monolayer was transfected with 1 μg of plasmid and transfected cells overexpressing LAMP-1 or shRNA for LAMP-1 were selected in G418 (50 μg/ml) or puromycin (5 μg/ml), respectively. Transfection of HeLa cells was verified by the expression of the reporter protein, green fluorescence protein (GFP), using florescence microscope as well as by immunoblotting using antibodies against human LAMP-1 (D401S; Cell Signaling Technology, Danvers, MA, USA).

### Immunoblotting

Cells were washed with cold PBS, and then lysed with lysis buffer with protease inhibitors as described before. The lysate was centrifuged at 10,000 × *g* for 15 min at 4 °C. The resulting supernatant was transferred to a new tube and protein was measured by Bradford assay. Total protein (25 μg) was resolved by SDS-PAGE and transferred to nitrocellulose membrane (Bio-Rad). The membrane was blocked in 5% skim milk for 45 min at room temperature followed by probing with primary antibodies overnight. After three washes with TBST (50 mM Tris-Cl, 150 mM NaCl, and 0.1% Tween 20; pH7.6), membrane was incubated with horseradish peroxidase (HRP)-conjugated secondary antibody for 2 h at room temperature and washed three times. Mouse anti-human LAMP-1 (D401S; Cell Signaling Technology) or rabbit anti-human LAMP-2 (PA1655, Thermo Fischer Scientific) or rabbit anti-human LAMP-3 (PA529566, Thermo Fischer Scientific) antibodies were used at 1:1000. The HRP-conjugated anti-mouse or anti-rabbit secondary antibodies (R&D systems, Minneapolis, MN, USA) were used at 1:1000. The luminescent signal was developed using Super signal substrate (Thermo Fischer Scientific) and images were captured using Gel-doc system (Bio-Rad).

### Immunochemistry

Cells were fixed with 3.7% paraformaldehyde in PBS for 30 min, washed twice with PBS, permeabilized and blocked for 30 min in 0.1% Triton X-100 (v/v)/1% bovine serum albumin (w/v) in PBS. All steps were conducted at room temperature. Permeabilized cells were incubated with primary antibodies at 4 °C for overnight followed by three washes with PBS and incubation with secondary antibodies for 2 h at room temperature and three more washes. Nuclei were stained with 2 μg/ml of Höechst 33342 (Thermo Fischer Scientific) for 5 min at room temperature followed by three washes and mounting. Mouse anti-human LAMP-1 (D401S; Cell Signaling Technology), rabbit anti-human LAMP-2 (PA1655, Thermo Fischer Scientific), and rabbit anti-human LAMP-3 (PA529566, Thermo Fischer Scientific) antibodies were used at 1:100. The fluorophore-conjugated CF488A goat anti-mouse or CF594 anti-rabbit secondary antibodies (Biotium, Fremont, CA, USA) were used at 1:1000. Images were acquired using a Nikon TE2000 wide field microscope with standard filter sets using ×10 objective and analyzed using Nikon image software. The distribution of lysosomes as a function of distance from the nucleus was determined by calculating the intensity of LAMP-1 fluorescence × area covered/number of nuclei in the field.

### Statistical analysis

Results are expressed as mean ± standard error of mean (S.E.M.). For comparisons, the statistical significance of differences in mean values was determined by analysis of variance (ANOVA) using GraphPad Prism 7 (GraphPad software, La Jolla, CA, USA). A *p*-value of 0.05 or less was considered statistically significant.

## Electronic supplementary material

Supplemental Table 1
