# Supplementary material for: Hydroxypropyl-beta and -gamma cyclodextrins rescue cholesterol accumulation in Niemann–Pick C1 mutant cell via lysosome-associated membrane protein 1
Source: Cell Death Dis. 2018 Oct 3;9(10):1019. doi: 10.1038/s41419-018-1056-1 (PMC6170477; doi:10.1038/s41419-018-1056-1)
Supplement: Supplementary file 1 — Supplemental Table 1 [file 41419_2018_1056_MOESM1_ESM.docx]

**Supplemental Table 1:**

Identification of cyclodextrin-regulated proteins using a mass-spectrometric shot-gun approach: Primary fibroblast cells from a healthy donor (wild type or WT) or NPC patient-derived fibroblasts were treated with HPβCD or HPγCD (1 mM, 72 h) and differentially expressed proteins were identified using LC-MS analysis (MudPIT), PEAKS8.0 and MyriMatch. Untreated WT fibroblasts were taken as a reference for analysis. A pairwise comparison was performed between WT vs. NPC + HPβCD; WT vs. NPC + HPγCD and NPC + HPβCD vs. NPC + HPγCD. The table 1a shows a list of 93 differentially expressed proteins in *NPC1^-/-^* cells common to both HPβCD and HPγCD treatments. The Table 1 b shows a list of 44 proteins exclusive to HPβCD and Table 1 c shows a list of 72 protein exclusively expressed under HPγCD treatment in *NPC1^-/-^* cells. The results are presented as fold expression change compared to WT. The cutoff value of fold protein expression change was set at > 2.0 or < 0.5 fold.

**Table 1 a:**

| **Accession ID** | **Name of the protein** | ***NPC1^-/-^* + HPβCD** | ***NPC1^-/-^* + HPγCD** |
| --- | --- | --- | --- |
| NP_006077 | tubulin beta-3 chain isoform 1 | 342.64 | 161.33 |
| NP_001276332 | MMS19 nucleotide excision repair protein homolog isoform 2 | 176.17 | 3.92 |
| NP_002618 | ATP-dependent 6-phosphofructokinase platelet type isoform 1 | 51.58 | 14.45 |
| XP_011531111 | PREDICTED: apolipoprotein B-100 isoform X1 | 48.91 | 23.03 |
| NP_001340 | aspartate--tRNA ligase cytoplasmic isoform 1 | 43.37 | 185.52 |
| NP_036265 | coatomer subunit gamma-2 isoform 1 | 37.26 | 12.39 |
| NP_620407 | mitogen-activated protein kinase 1 | 30.16 | 4.55 |
| NP_001137290 | plasminogen activator inhibitor 2 | 25.23 | 8.1 |
| NP_002787 | proteasome subunit beta type-4 | 21.33 | 9.59 |
| NP_001018083 | phosphoenolpyruvate carboxykinase [GTP] mitochondrial isoform 2 precursor | 19.43 | 3.63 |
| NP_001020419 | deoxyuridine 5'-triphosphate nucleotidohydrolase mitochondrial isoform 1 precursor | 16.58 | 3.7 |
| NP_863656 | cytosolic acyl coenzyme A thioester hydrolase isoform hBACHd | 15.25 | 4.38 |
| NP_001266289 | acyl-protein thioesterase 1 isoform 6 | 13.32 | 35.74 |
| NP_006699 | lactoylglutathione lyase | 11.34 | 3.35 |
| NP_001121188 | electron transfer flavoprotein subunit alpha mitochondrial isoform b | 11.03 | 3.67 |
| NP_579899 | myoferlin isoform b | 10.59 | 4.08 |
| XP_005252505 | PREDICTED: integrin beta-1 isoform X1 | 10.45 | 21.28 |
| NP_000173 | trifunctional enzyme subunit alpha mitochondrial precursor | 9.33 | 3.02 |
| NP_071908 | nucleoredoxin isoform 1 | 8.51 | 21.85 |
| NP_002583 | proliferating cell nuclear antigen | 8.3 | 0.08 |
| XP_011512065 | PREDICTED: protein NOXP20 isoform X2 | 8.14 | 2.75 |
| XP_011533816 | PREDICTED: elongation factor 1-alpha 1 isoform X1 | 7.55 | 18.5 |
| XP_005249975 | PREDICTED: secernin-1 isoform X1 | 7.26 | 3.62 |
| NP_066964 | X-ray repair cross-complementing protein 5 | 7.25 | 0.31 |
| XP_011516725 | PREDICTED: proteasome-associated protein ECM29 homolog isoform X5 | 7.24 | 88.65 |
| NP_057381 | prenylcysteine oxidase 1 precursor | 6.94 | 29.6 |
| NP_001265568 | ran-specific GTPase-activating protein isoform 1 | 6.71 | 2.93 |
| NP_003745 | eukaryotic translation initiation factor 3 subunit F | 6.24 | 29.74 |
| XP_005269696 | PREDICTED: cytosolic purine 5'-nucleotidase isoform X7 | 5.99 | 0.41 |
| NP_001227 | carbonyl reductase [NADPH] 3 | 5.95 | 23.42 |
| NP_001279 | chloride intracellular channel protein 1 | 5.21 | 2.19 |
| NP_000009 | very long-chain specific acyl-CoA dehydrogenase mitochondrial isoform 1 precursor | 5.12 | 24.35 |
| NP_039234 | chloride intracellular channel protein 4 | 5.11 | 2.34 |
| XP_011519231 | PREDICTED: peptidyl-prolyl cis-trans isomerase FKBP4 isoform X1 | 4.58 | 0.45 |
| NP_006358 | adenylyl cyclase-associated protein 1 | 4.46 | 9.98 |
| XP_011542434 | PREDICTED: fumarate hydratase mitochondrial isoform X1 | 4.19 | 2.05 |
| NP_001287921 | eukaryotic translation initiation factor 3 subunit K isoform 2 | 3.65 | 0.34 |
| NP_066977 | phosphoserine aminotransferase isoform 2 | 3.64 | 0.31 |
| NP_000980 | 60S ribosomal protein L30 | 3.56 | 9.95 |
| NP_443198 | protein Niban | 3.43 | 0.34 |
| NP_006504 | serine--tRNA ligase cytoplasmic | 3.42 | 0.42 |
| NP_003741 | eukaryotic translation initiation factor 3 subunit A | 3.38 | 11.51 |
| XP_011534986 | PREDICTED: ERO1-like protein alpha isoform X1 | 2.95 | 7.37 |
| NP_005827 | ribonuclease UK114 | 2.93 | 42.42 |
| XP_006711978 | PREDICTED: leucine-rich PPR motif-containing protein mitochondrial isoform X2 | 2.92 | 25.1 |
| NP_009215 | twinfilin-2 | 2.75 | 0.36 |
| NP_001814 | creatine kinase B-type | 2.51 | 0.13 |
| XP_005265154 | PREDICTED: acylamino-acid-releasing enzyme isoform X6 | 2.45 | 5.53 |
| NP_002547 | oxysterol-binding protein 1 | 2.38 | 8.88 |
| NP_001157791 | filamin-B isoform 4 | 2.37 | 0.11 |
| NP_001263218 | cAMP-dependent protein kinase type I-alpha regulatory subunit isoform a | 2.37 | 0.01 |
| NP_001180546 | non-specific lipid-transfer protein isoform 8 proprotein | 2.36 | 8.02 |
| NP_009057 | transitional endoplasmic reticulum ATPase | 2.35 | 4.89 |
| NP_006746 | transaldolase | 2.25 | 0.18 |
| NP_006382 | importin-7 | 2.17 | 42.99 |
| NP_001269429 | tetratricopeptide repeat protein 1 | 2.17 | 7.42 |
| XP_005246524 | PREDICTED: glutaminase kidney isoform mitochondrial isoform X2 | 2.08 | 0.47 |
| NP_067072 | sorting nexin-6 isoform a | 2.06 | 0.04 |
| NP_001289976 | myosin regulatory light chain 12A isoform 1 | 2.04 | 0.39 |
| NP_000745 | catechol O-methyltransferase isoform MB-COMT | 0.48 | 12.49 |
| NP_004451 | prolyl endopeptidase FAP isoform 1 | 0.43 | 7.38 |
| NP_001687 | V-type proton ATPase subunit E 1 isoform a | 0.43 | 0.02 |
| NP_009209 | gamma-aminobutyric acid receptor-associated protein | 0.41 | 0.1 |
| NP_821067 | serine/threonine-protein phosphatase 2A activator isoform b | 0.39 | 0.16 |
| NP_001243572 | leukotriene A-4 hydrolase isoform 2 | 0.39 | 0.02 |
| XP_005267356 | PREDICTED: protein AHNAK2 isoform X1 | 0.38 | 0.07 |
| NP_001275959 | N-acetylgalactosamine kinase isoform 3 | 0.35 | 2.09 |
| NP_001091974 | probable ATP-dependent RNA helicase DDX17 isoform 3 | 0.32 | 0.06 |
| XP_006717377 | PREDICTED: far upstream element-binding protein 3 isoform X7 | 0.29 | 0.03 |
| NP_005042 | glutamine--tRNA ligase isoform a | 0.26 | 2.9 |
| NP_002619 | profilin-2 isoform b | 0.25 | 0.05 |
| NP_009035 | 60S ribosomal protein L10a | 0.24 | 0.12 |
| XP_011529432 | PREDICTED: filamin-A isoform X4 | 0.24 | 0.07 |
| NP_001129602 | amyloid beta A4 protein isoform f precursor | 0.21 | 0.1 |
| NP_004172 | ubiquitin carboxyl-terminal hydrolase isozyme L1 | 0.21 | 0.01 |
| NP_001030168 | 60S ribosomal protein L14 | 0.18 | 0.01 |
| XP_006721609 | PREDICTED: phosphoribosylformylglycinamidine synthase isoform X2 | 0.11 | 0.27 |
| XP_005254668 | PREDICTED: sorting nexin-1 isoform X1 | 0.11 | 0.25 |
| NP_006127 | F-actin-capping protein subunit alpha-2 | 0.11 | 0.22 |
| NP_055416 | EH domain-containing protein 2 | 0.1 | 0.25 |
| NP_001075109 | DNA-dependent protein kinase catalytic subunit isoform 2 | 0.07 | 0.01 |
| XP_005260463 | PREDICTED: glutathione synthetase isoform X1 | 0.06 | 0.27 |
| NP_001238979 | protein-L-isoaspartate(D-aspartate) O-methyltransferase isoform 3 | 0.05 | 0.32 |
| XP_006712428 | PREDICTED: heterogeneous nuclear ribonucleoprotein A3 isoform X3 | 0.04 | 0.32 |
| NP_115679 | hydroxysteroid dehydrogenase-like protein 2 isoform 1 | 0.03 | 0.19 |
| NP_000973 | 60S ribosomal protein L21 | 0.03 | 0.01 |
| XP_005257851 | PREDICTED: hepatocyte growth factor-regulated tyrosine kinase substrate isoform X2 | 0.02 | 0.07 |
| NP_001189333 | alpha-aminoadipic semialdehyde dehydrogenase isoform 3 | 0.01 | 0.14 |
| NP_001106177 | adseverin isoform 1 | 0.01 | 0.1 |
| NP_003008 | serine/arginine-rich splicing factor 3 | 0.01 | 0.05 |
| XP_011523798 | PREDICTED: puromycin-sensitive aminopeptidase isoform X1 | 0.01 | 0.03 |
| NP_002096 | histone H2AX | 0.01 | 0.03 |
| NP_620164 | carboxymethylenebutenolidase homolog | 0.01 | 0.02 |

**Table 1 b:**

| **Accession ID** | **Name of the protein** | ***NPC1^-/-^* + HPβCD** | ***NPC1^-/-^* + HPγCD** |
| --- | --- | --- | --- |
| NP_002152 | isoleucine--tRNA ligase cytoplasmic | 4.53 | 1.56 |
| NP_002769 | prosaposin isoform a preproprotein | 4.13 | 1.77 |
| NP_004896 | peroxiredoxin-6 | 4.11 | 1.69 |
| NP_001269581 | stress-induced-phosphoprotein 1 isoform a | 3.54 | 0.96 |
| NP_057212 | coatomer subunit gamma-1 | 3.33 | 1.51 |
| XP_005275347 | PREDICTED: valine--tRNA ligase isoform X1 | 3.32 | 0.88 |
| NP_258412 | inosine triphosphate pyrophosphatase isoform a | 3.04 | 1.11 |
| XP_011539896 | PREDICTED: procollagen-lysine 2-oxoglutarate 5-dioxygenase 1 isoform X2 | 2.85 | 1.12 |
| NP_002617 | ATP-dependent 6-phosphofructokinase liver type isoform b | 2.84 | 0.96 |
| NP_055362 | tropomodulin-3 | 2.77 | 0.99 |
| NP_005989 | T-complex protein 1 subunit gamma isoform a | 2.58 | 0.62 |
| NP_036611 | 14-3-3 protein gamma | 2.57 | 0.53 |
| XP_005253351 | PREDICTED: serine-threonine kinase receptor-associated protein isoform X1 | 2.51 | 0.68 |
| NP_001129667 | transportin-2 isoform 2 | 2.43 | 1.13 |
| NP_001186797 | glutaredoxin-3 | 2.36 | 1.14 |
| NP_003350 | UDP-glucose 6-dehydrogenase isoform 1 | 2.34 | 0.91 |
| NP_001651 | ADP-ribosylation factor 4 | 2.31 | 1.01 |
| NP_000393 | glucose-6-phosphate 1-dehydrogenase isoform a | 2.15 | 0.84 |
| XP_011522182 | PREDICTED: myosin-10 isoform X7 | 2.15 | 0.56 |
| XP_005254502 | PREDICTED: pyruvate kinase PKM isoform X1 | 2.06 | 0.75 |
| NP_001189485 | CD44 antigen isoform 7 precursor | 0.48 | 1.08 |
| XP_011519410 | PREDICTED: tripeptidyl-peptidase 2 isoform X5 | 0.38 | 1.15 |
| NP_004795 | probable cytosolic iron-sulfur protein assembly protein CIAO1 | 0.38 | 1.08 |
| NP_066272 | PDZ and LIM domain protein 1 | 0.38 | 0.85 |
| NP_003339 | ubiquitin-conjugating enzyme E2 N | 0.38 | 0.77 |
| XP_005264603 | PREDICTED: exportin-1 isoform X5 | 0.36 | 0.9 |
| NP_001287673 | protein transport protein Sec31A isoform 6 | 0.34 | 1.11 |
| NP_004578 | ribosome-binding protein 1 | 0.32 | 0.78 |
| NP_006614 | D-3-phosphoglycerate dehydrogenase | 0.3 | 1.28 |
| NP_001011 | 40S ribosomal protein S16 | 0.27 | 0.6 |
| XP_006712195 | PREDICTED: translation initiation factor eIF-2B subunit delta isoform X2 | 0.25 | 0.74 |
| NP_006182 | proliferation-associated protein 2G4 | 0.2 | 1.74 |
| NP_002699 | serine/threonine-protein phosphatase PP1-alpha catalytic subunit isoform 1 | 0.2 | 1.28 |
| NP_000683 | aldehyde dehydrogenase X mitochondrial precursor | 0.15 | 1.16 |
| NP_000081 | collagen alpha-1(III) chain preproprotein | 0.14 | 0.52 |
| NP_001018006 | tropomyosin alpha-1 chain isoform Tpm1.7cy | 0.13 | 0.67 |
| NP_001186271 | 60S ribosomal protein L17 isoform a | 0.12 | 1.53 |
| NP_006827 | translational activator GCN1 | 0.11 | 1.08 |
| NP_001185709 | actin-related protein 2/3 complex subunit 4 isoform c | 0.08 | 0.78 |
| NP_057164 | glyoxalase domain-containing protein 4 | 0.07 | 1.05 |
| NP_067643 | PDZ and LIM domain protein 2 isoform 2 | 0.06 | 0.99 |
| NP_037466 | mannose-1-phosphate guanyltransferase beta isoform 1 | 0.04 | 0.97 |
| XP_011507514 | PREDICTED: BRO1 domain-containing protein BROX isoform X2 | 0.04 | 0.65 |
| XP_011518435 | PREDICTED: nucleobindin-2 isoform X5 | 0.01 | 1.39 |

**Table 1 c:**

| **Accession ID** | **Name of the protein** | ***NPC1^-/-^* + HPβCD** | ***NPC1^-/-^* + HPγCD** |
| --- | --- | --- | --- |
| NP_000427 | succinyl-CoA:3-ketoacid coenzyme A transferase 1 mitochondrial precursor | 1 | 130.02 |
| NP_060238 | leucine-rich repeat-containing protein 40 | 1 | 31.85 |
| XP_011509821 | PREDICTED: prothymosin alpha isoform X2 | 1 | 17.25 |
| NP_005336 | heat shock 70 kDa protein 1A/1B | 0.98 | 14.89 |
| XP_011519775 | PREDICTED: aminopeptidase N isoform X1 | 1.68 | 13.42 |
| XP_011524374 | PREDICTED: cytosolic non-specific dipeptidase isoform X1 | 1.97 | 9.18 |
| NP_057226 | very-long-chain 3-oxoacyl-CoA reductase | 1 | 8.68 |
| NP_000254 | alpha-N-acetylglucosaminidase precursor | 0.86 | 7.7 |
| NP_001119 | AP-1 complex subunit gamma-1 isoform b | 1 | 7.42 |
| NP_004517 | DNA replication licensing factor MCM2 | 1 | 7.08 |
| NP_003935 | selenium-binding protein 1 isoform 1 | 0.96 | 6.93 |
| NP_001003794 | monoglyceride lipase isoform 2 | 1 | 6.43 |
| NP_056991 | cytosol aminopeptidase | 1.28 | 6.33 |
| XP_011527230 | PREDICTED: protein NDRG3 isoform X6 | 1.59 | 6.14 |
| NP_001122384 | poly(rC)-binding protein 2 isoform e | 1 | 5.92 |
| NP_004773 | synaptosomal-associated protein 29 | 1 | 5.72 |
| NP_002799 | 26S proteasome non-ATPase regulatory subunit 2 isoform 1 | 1.74 | 5.54 |
| NP_001268442 | trifunctional enzyme subunit beta mitochondrial isoform 3 | 1 | 5.39 |
| XP_011513781 | PREDICTED: tensin-3 isoform X5 | 1 | 5.3 |
| NP_054745 | m7GpppX diphosphatase | 1 | 5.24 |
| NP_001004 | 40S ribosomal protein S9 | 1 | 4.05 |
| NP_001275899 | acyl-CoA synthetase family member 2 mitochondrial isoform 4 | 1 | 3.94 |
| XP_005246695 | PREDICTED: peptidyl-prolyl cis-trans isomerase FKBP7 isoform X1 | 1 | 3.94 |
| XP_011533095 | PREDICTED: golgin subfamily A member 3 isoform X3 | 1.72 | 3.89 |
| NP_057086 | saccharopine dehydrogenase-like oxidoreductase | 1 | 3.85 |
| NP_005552 | lysosome-associated membrane glycoprotein 1 precursor | 1.69 | 3.71 |
| NP_001273063 | synembryn-A isoform 2 | 1 | 3.62 |
| NP_065843 | neutral cholesterol ester hydrolase 1 isoform b | 0.51 | 3.45 |
| NP_002878 | arginine--tRNA ligase cytoplasmic | 1.61 | 3.26 |
| XP_006716162 | PREDICTED: synaptophysin-like protein 1 isoform X1 | 1 | 3.1 |
| XP_005272276 | PREDICTED: nuclear pore complex protein Nup214 isoform X6 | 1 | 2.83 |
| XP_006713048 | PREDICTED: catenin beta-1 isoform X3 | 1 | 2.76 |
| NP_064601 | aminopeptidase B | 0.82 | 2.54 |
| NP_001257329 | tubulin alpha-1A chain isoform 2 | 1 | 2.48 |
| XP_011534922 | PREDICTED: E3 ubiquitin-protein ligase HECTD1 isoform X4 | 1 | 2.45 |
| XP_005259774 | PREDICTED: acetolactate synthase-like protein isoform X1 | 1 | 2.36 |
| NP_060810 | serine/threonine-protein phosphatase CPPED1 isoform a | 1 | 2.19 |
| XP_011521840 | PREDICTED: fatty acid synthase isoform X1 | 1 | 2.12 |
| NP_055077 | prolyl 3-hydroxylase 3 precursor | 1 | 2.09 |
| XP_011538832 | PREDICTED: nuclear migration protein nudC isoform X2 | 1 | 2.06 |
| NP_659477 | protein kinase C delta-binding protein | 1 | 2.04 |
| NP_002794 | 26S protease regulatory subunit 7 isoform 1 | 1 | 0.49 |
| XP_005251291 | PREDICTED: DNA replication licensing factor MCM4 isoform X1 | 1 | 0.47 |
| NP_005372 | nucleolin | 1.65 | 0.47 |
| NP_001273689 | probable inactive glycosyltransferase 25 family member 3 isoform b | 1 | 0.46 |
| NP_001989 | fibulin-2 isoform b precursor | 1 | 0.45 |
| NP_003396 | 14-3-3 protein eta | 1.7 | 0.42 |
| NP_001060 | tubulin beta-2A chain | 0.8 | 0.4 |
| XP_005275426 | PREDICTED: guanine nucleotide-binding protein-like 1 isoform X1 | 0.97 | 0.39 |
| NP_065392 | adipocyte plasma membrane-associated protein | 1 | 0.35 |
| NP_006657 | ruvB-like 2 | 1.38 | 0.34 |
| NP_002558 | phosphatidylethanolamine-binding protein 1 preproprotein | 0.71 | 0.31 |
| NP_004981 | methionine--tRNA ligase cytoplasmic | 1.22 | 0.3 |
| NP_001230059 | 60S ribosomal protein L13 isoform 2 | 1 | 0.28 |
| NP_001155238 | adenosylhomocysteinase isoform 2 | 1.27 | 0.28 |
| NP_001419 | alpha-enolase isoform 1 | 1.29 | 0.27 |
| NP_002563 | platelet-activating factor acetylhydrolase IB subunit beta isoform a | 0.99 | 0.26 |
| XP_006714926 | PREDICTED: heterogeneous nuclear ribonucleoprotein H isoform X4 | 0.7 | 0.23 |
| NP_001748 | carbonyl reductase [NADPH] 1 isoform 1 | 1.47 | 0.22 |
| NP_003133 | lupus La protein | 1.19 | 0.17 |
| NP_001914 | DNA damage-binding protein 1 | 0.73 | 0.17 |
| XP_005266757 | PREDICTED: asparagine--tRNA ligase cytoplasmic isoform X1 | 1.45 | 0.17 |
| NP_001184223 | dihydropyrimidinase-related protein 3 isoform 1 | 0.89 | 0.16 |
| XP_011537870 | PREDICTED: WASH complex subunit FAM21C isoform X3 | 0.95 | 0.14 |
| NP_057417 | serine/arginine repetitive matrix protein 2 | 1.28 | 0.14 |
| NP_001988 | ubiquitin-like protein fubi and ribosomal protein S30 precursor | 0.63 | 0.13 |
| NP_054891 | 14 kDa phosphohistidine phosphatase isoform 3 | 0.85 | 0.13 |
| NP_004308 | ATPase ASNA1 | 1 | 0.12 |
| NP_001406 | eukaryotic translation initiation factor 2 subunit 3 | 0.6 | 0.12 |
| NP_937885 | eukaryotic translation initiation factor 4 gamma 1 isoform 3 | 0.66 | 0.1 |
| NP_001240312 | 60S ribosomal protein L15 isoform 1 | 0.58 | 0.08 |
| NP_000975 | 60S ribosomal protein L23a | 0.85 | 0.07 |
| NP_036437 | AP-2 complex subunit alpha-2 isoform 2 | 1.61 | 0.07 |
| NP_036382 | ras-related protein R-Ras2 isoform a | 1 | 0.05 |
| NP_001254738 | interleukin enhancer-binding factor 2 isoform 2 | 0.52 | 0.05 |
| XP_011528279 | PREDICTED: adenylosuccinate lyase isoform X2 | 1.71 | 0.04 |
| NP_001257356 | 26S proteasome non-ATPase regulatory subunit 5 isoform 2 | 0.77 | 0.02 |
| NP_001257411 | 26S proteasome non-ATPase regulatory subunit 11 | 0.82 | 0.01 |
| XP_011522757 | PREDICTED: AP-2 complex subunit beta isoform X5 | 0.6 | 0.01 |
